# Role of cultivation media in the development of yeast strains for large scale industrial use

**DOI:** 10.1186/1475-2859-4-31

**Published:** 2005-11-10

**Authors:** Bärbel Hahn-Hägerdal, Kaisa Karhumaa, Christer U Larsson, Marie Gorwa-Grauslund, Johann Görgens, Willem H van Zyl

**Affiliations:** 1 Applied Microbiology, LTH/Lund University, P O Box 124, SE-221 00 Lund, Sweden; 2 Department of Process Engineering, Stellenbosch University, Private Bag X1, Stellenbosch, 7602, South Africa; 3 Department of Microbiology, Stellenbosch University, Private Bag X1, Stellenbosch

## Abstract

The composition of cultivation media in relation to strain development for industrial application is reviewed. Heterologous protein production and pentose utilization by *Saccharomyces cerevisiae *are used to illustrate the influence of media composition at different stages of strain construction and strain development. The effects of complex, defined and industrial media are compared. Auxotrophic strains and strain stability are discussed. Media for heterologous protein production and for bulk bio-commodity production are summarized.

## Introduction

The composition of the medium used for cultivation of micro-organisms is directly reflected in their physiological phenotype and their fermentation performance, which in turn affects the results of strain analyses and strain performance in industrial applications. For this reason, the successful development of strains for large scale industrial production of heterologous proteins [1,2] and low-value fuels, chemicals and materials [3,4] merits the composition of cultivation media in various steps of strain development to be reconsidered.

Introducing novel recombinant strains into industrially relevant cultivation media may reveal that the strain has not been properly designed for this environment. For example, it was found that a strain of the lactic acid bacterium *Streptococcus thermophilus *engineered for enhanced exopolysaccharide production – a trait highly desirable in yoghurt production – failed to express the phenotype in milk without the addition of an extra nitrogen source [5]. Similarly, a genetically modified strain of the yeast *Saccharomyces cerevisiae*, which had been communicated as the ultimate solution to the fermentation of lignocellulose derived xylose [6,7], was found to require yeast extract, additional hexose sugar and oxygenation to efficiently ferment the xylose fraction in spent sulphite liquor [8]. Furthermore, heterologous protein production in yeast is strongly influenced by the nitrogen-composition of the production medium [9,10]. Thus the final industrial environment must be considered throughout the strain development process to avoid unfounded expectation and – more importantly – to prevent costly investment into premature production facilities.

A cultivation medium is designed to reflect the elemental composition and the biosynthetic capacity of a given microbial cell (see e.g. [11]). While the elemental composition of microbial cells is relatively similar, their biosynthetic capacity varies widely. The yeast *S. cerevisiae *and the bacterium *Escherichia coli *have extensive biosynthetic capacity and grow in defined mineral media [12]. In contrast, the biosynthetic capacity of many lactic acid bacteria is limited and they require rich or extensively supplemented medium for efficient growth [13]. Furthermore, economic constraints make the large-scale production of low-cost products reliant on cheap sources of carbon and nitrogen, such as molasses from the sugar industry, corn steep liquor from the starch industry, spent sulphite liquor from the forest products industry and cheese whey from the dairy industry [14,15]. In addition to providing carbon, nitrogen, vitamins and trace elements necessary for cell growth and metabolite production, such industrial media may also contain substances which inhibit growth and metabolite production.

This paper reviews the influence of the composition of cultivation media on the development of novel industrial production strains with the view that it is necessary to consider the final cultivation conditions in every stage of strain development. Primarily two types of recombinant strains of *S. cerevisiae *are used as examples: strains which produce heterologous proteins and strains with an expanded substrate range to include pentose sugars. Experience from other organisms is included to complement the discussion. Finally, genetic engineering approaches to overcome industrial media constraints are also exemplified.

### Metabolic engineering, evolutionary engineering and systems biology in strain development

**Figure 1:**
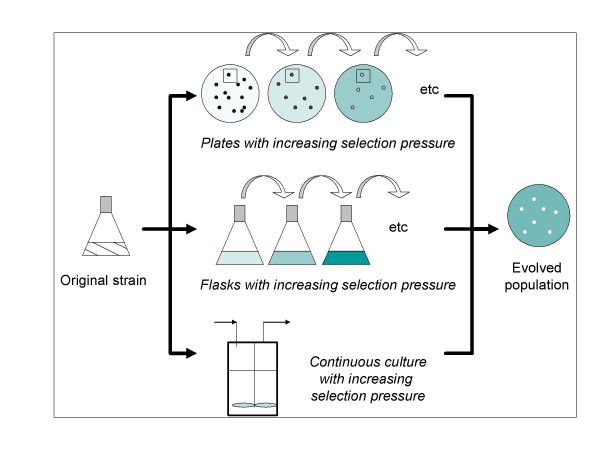
Principle of Evolutionary Engineering [18-20].

**Figure 2:**
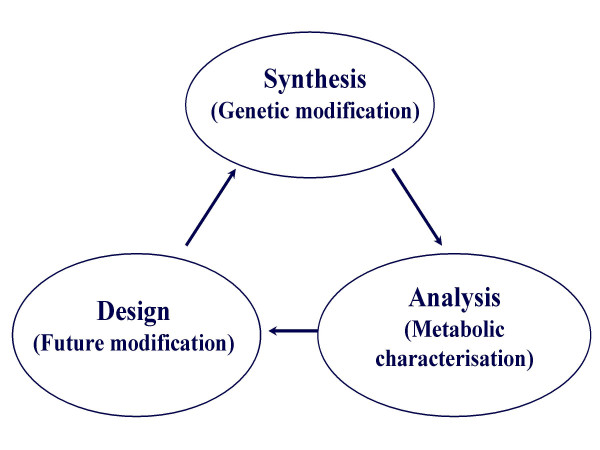
Principe of Metabolic Engineering (adapted from [23]).

Traditionally, novel production strains have been developed by mutagenesis [16], breeding [17], and the lately revived concept of evolutionary engineering (Figure 1; [18-20]). Strains with novel traits are now also developed by life science technologies including genetic and metabolic engineering (Figure 2; [21-23]). In recent years these engineering concepts have been expanded in the context of systems biology to also include information and system science technologies [24-26]. In metabolic engineering, cells are iteratively analyzed, designed and synthesized (Figure 2) using molecular tools such as recombinant DNA technology and genomic information, [27,28]. Evolutionary engineering (Figure 1) relies on carefully designed selection protocols, i. e. media and cultivation conditions [18-20] for the development of strains with industrially interesting characteristics. Metabolic and evolutionary engineering technologies may also be combined to generate novel traits [29-33]. The multitude of data generated in the analysis of the genome, transcriptome, proteome and metabolome [25] requires the use of information and system science technologies to translate these data into design strategies for next rounds of metabolic and evolutionary engineering [24]. Several studies have pointed out that the cultivation conditions and media composition used for the analysis of novel engineered strains strongly influence the data generated [34-39]. Since such data form the basis for the design strategy for the following rounds of strain development, it is evident that choice of cultivation media is a fundamental and integral part of strain development.

### Media and strain stability

Whereas strain development by recombinant techniques is usually performed in genetically defined laboratory strains harboring markers suitable for selection of transformed cells in chemically defined cultivation media, the typical industrial production strain is genetically undefined and adapted to perform in rather poor, toxic, viscous, and nutrient-limited media. Once desired novel traits have been established in recombinant laboratory strains, the novel strains are either directly transferred to the industrial production environment or – as occurs much more frequently – a potential production strain has to undergo a new round of metabolic engineering procedures. In both cases, the medium in which the novel pathways are developed differs substantially from the medium in which the final production strain is expected to perform.

The genetic stability of strains is an absolute requirement for utilization in industrial processes. Due to the adaptive nature of microorganisms, attention should also be directed towards the stability of any novel traits in recombinant or mutant strains. In industry, rich or undefined media are often used, which may result in unexpected loss of plasmids and even chromosomal modifications. Prolonged cultivation, for example in continuous fermentation set-ups, increases the probability of detrimental genetic instability. Even in mineral medium, loss of plasmids with auxotrophic marker has been reported in prolonged continuous cultures [40]. This was caused by released amino acids from the dying cells, and probably also by spontaneous chromosomal insertion of the marker gene [40].

In *S. cerevisiae*, both episomal plasmids (YEp; [41]) and integrative plasmids (YIp; [42]) are used as expression vectors for heterologous protein expression and metabolic engineering. The advantage of YIp vectors, despite their low copy numbers, is their robust genetic stability even in unselective medium due to the integration of the vector into the yeast genome [43-51]. The benefit of using YEp plasmids is the high gene copy number of up to 70 copies per cell [52] resulting in high expression levels of the desired proteins, although their high segregational instability often results in plasmid loss especially in rich medium [53,54]. However, the stability of YEp-type vectors can be improved by autoselection systems, such as the *fur1 ura3 *system [55], where the deletion of *FUR1 *together with the use of a plasmid containing the *URA3 *marker results in stable plasmid expression even in continuous culture [56]. Without such autoselection systems it is necessary to use a selective medium to overcome the instability of YEp plasmids, which may pose a limitation to the industrial use of such strains especially with low-cost products.

### Rich complex media versus defined media

Generally, microorganisms grow more vigorously in rich media than in mineral media, because rich media contain biosynthetic precursors that can be channeled directly into anabolic pathways, reducing the need to produce biosynthetic precursors and saving metabolic energy. This has a significant effect on growth and production characteristics.

For example, a three-fold increase in production levels of heterologous laccase by recombinant *Yarrowia lipolytica *was reported when switching from yeast nitrogen base (YNB) to complex medium [10]. When autoselective strains of *S. cerevisiae *expressing heterologous xylanase or α-L-arabinofuranosidase genes were cultivated in complex YPD medium, 24-fold higher xylanase and up to 70-fold higher levels of α-L-arabinofuranosidase were produced [57,58]. Similarly, production levels of the potent thrombin-specific inhibitor, hirudin, by recombinant *S. cerevisiae *was improved 20 fold in complex medium [59], demonstrating the substantial impact of medium composition on heterologous protein production.

Aoki et al. [60] elegantly demonstrated single-step purification of recombinant cysteine proteinase (NsCys) from *Pichia pastoris *by switching medium composition during cultivation. The recombinant *P. pastoris *was first cultivated in glycerol complex medium to generate biomass in a short time. The cells were harvested and resuspended in minimal medium for induction of NsCys production. The minimal medium faciliated protein secretion and subsequent purification.

Strains of *E. coli *with altered levels of pyruvate decarboxylase and alcohol dehydrogenase displayed a reduced flux of pyruvate into the native fermentation pathways when cultivated in defined medium [61]. In addition, the flow of carbon skeletons into the 2-ketoglutarate arm of the tricarboxylic acid pathway and biosynthesis was restricted, which dramatically reduced growth yields in defined medium compared with complex medium. The observations demonstrated that inherent limitations in the metabolism of engineered strains can be masked by the presence of complex nutrients in the medium and are often not observed without cultivation in defined medium.

To illustrate the influence of media composition on strain performance, we compared the growth of baker's yeast and two recombinant strains: a laboratory strain of *S. cerevisiae*, TMB3001 [62] and an industrial strain of *S. cerevisiae*, TMB3400 [29]. Both recombinant strains have been engineered for xylose utilization with the introduction of the *XYL1 *[63] and *XYL2 *[64] genes encoding xylose reductase and xylitol dehydrogenase, respectively, from the yeast *Pichia stipitis*. In addition the endogenous gene *XKS1 *[65] encoding xylulokinase has been overexpressed.

We evaluated the influence of four commonly used media on growth and product formation under aerobic and oxygen limited conditions: yeast extract-peptone (YP [66,67]); a defined mineral medium (DM; [68]); yeast nitrogen base (YNB; [12]); and synthetic complete (SC) medium equivalent to supplemented YNB [12] (Table 1). YP is an undefined rich complex medium composed of yeast extract (YE) and peptone. YE is prepared by autolysis of whole yeast cells at around 50°C [66,69-71] and peptone is an acid- or enzymatic hydrolysate of a protein-rich by-product from the food and feed industry [67]. YP contains all components necessary for propagation of yeast cells, including biosynthetic building blocks, and it is frequently used in the initial stages of fermentation when a large inoculum is required. YNB is a chemically defined medium that can be supplemented to satisfy auxotrophic requirements of yeast mutants used in metabolic engineering, then referred to as SC medium. DM medium contains almost all components of YNB medium (Table 1), however, some components are present in higher and even an order of magnitude higher concentration than in YNB medium. The DM medium and variants thereof are commonly used to obtain quantitative physiological data for yeast strains. It has been designed to assure that concentrations of vitamins and trace elements do not exercise growth limitation [68]. Sodium chloride, riboflavin and folic acid were not found to be necessary for growth of *S. cerevisiae*, whereas cobalt apparently supported growth (Table 1). EDTA seems to be required to dissolve elevated concentrations of trace elements.

**Figure 3:**
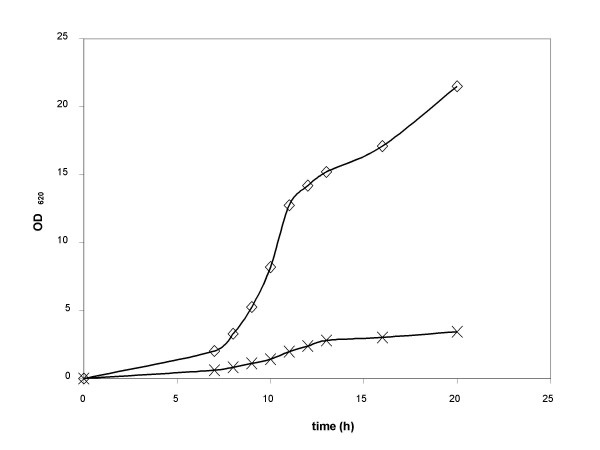
Aerobic growth of baker's yeast in YP medium buffered with 50 mM Phthalate. With glucose -◊- (μmax = 0.45), Without glucose -×- (μmax = 0.29)

YP supported growth of a commercial baker's yeast strain even in the absence of additional carbon source (Figure 3). The maximum specific growth rate under these conditions was 0.29 h^-1 ^with a final OD_620 _of 3–4 after 24 hours. With additional 20 g/l glucose an OD_620 _of 22 was reached at a maximum specific growth rate of 0.45 h^-1^. Sugars present in YE may explain this phenomenon. Yeast accumulates storage carbohydrates such as glycogen and trehalose, the amount of which is strongly dependent on cultivation conditions [72]. During the preparation of YE these compounds are fully or partially hydrolyzed to monomer glucose. YE also contains lactate, which can serve as carbon source in yeast cultivation [9]. Lactate is a consequence of non-sterile cultivation conditions in baker's and brewer's yeast production [73]. In addition to these auxiliary carbon sources, YE also contains a number of other compounds, which strongly influence fermentation performance [9].

The maximum specific growth rate of TMB3001 and TMB3400 in the four different media (Table 2) under aerobic and oxygen limited conditions varied considerably. Highest growth rates were obtained in aerobic YP medium and significantly lower growth rates were observed in the three defined media both for glucose and xylose as carbon source (Table 2). The results support previous observations that complex media components can mask inherent limitations in the metabolism of recombinant strains as demonstrated for *E. coli *[61] and *S. cerevisiae *[8].

In all media TMB3400 displayed significantly higher specific growth rate on xylose than TMB3001 confirming previous results [29,32]. Reducing the oxygen supply emphasized this difference. Both strains displayed significantly lower specific growth rate on xylose than on glucose (Table 2) confirming previous observations [29]. Also in this comparison, oxygen limitation increased the difference. DM and YNB medium resulted in almost identical specific growth rates indicating that the level of trace elements and vitamins in YNB were not limiting growth under the presently chosen cultivation conditions. Amino acid supplementation in YNB slightly increased the specific growth rates during xylose utilization but not under glucose utilization (Table 2).

**Figure 4:**
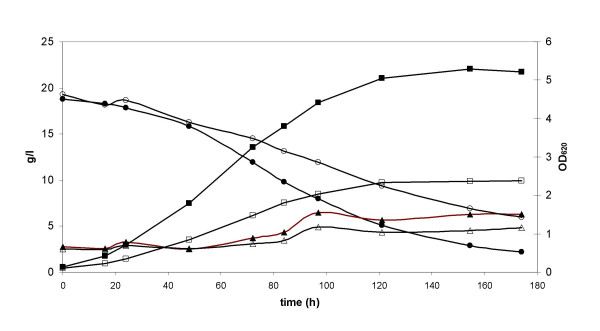
Xylose utilisation and product formation during oxygen-limited cultivation of TMB 3400 [29] in YP and YNB media. Xylose YP-●-, Xylose YNB -○-, Ethanol YP -▴-, Ethanol YNB -△-, OD_620 _YP -■-, OD_620 _YNB -□- Sugars and products were analyzed with HPLC (Bio-Rad, Aminex 87-H column).

The volumetric xylose uptake mirrored the growth rate in all four media with both strains and under the two levels of oxygenation (Table 2). Figure 4 shows the time course for xylose consumption and product formation for TMB3400 under oxygen limited conditions using YP and YNB medium. Results from only one of the mineral media are displayed since growth and product formation was identical in all three mineral media. Whereas the results emphasize the strong growth promoting influence of the YP medium they also show that the media composition did not influence the distribution of products under the chosen conditions (Figure 4).

### Buffers

**Figure 5:**
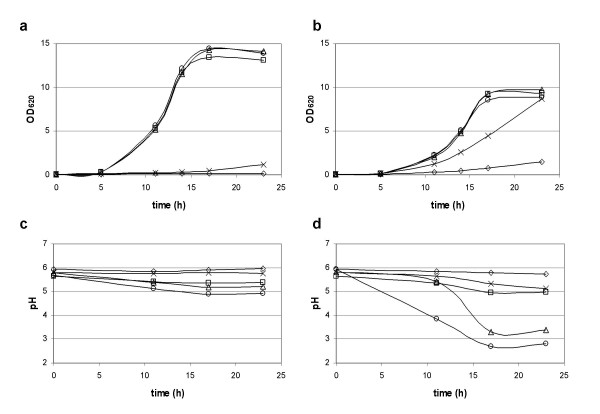
a-d. Influence of 50 mM buffer on aerobic growth of TMB 3001 [62] in YP and DM media. a, c: glucose 20 g/l in YP, b, d: glucose 20 g/l in DM. Non buffered -○-, Phthalate -□-, Phosphate -△-, Citrate-Phosphate -×-, Citrate -◊-

Strain development may require large numbers of strains to be evaluated for their performance in simple screening set-ups [74,75], where neither oxygen availability nor pH is controlled. When microorganisms grow in defined mineral medium with ammonium as the sole nitrogen source, the medium quickly acidifies due to proton excretion during active transport of nutrients into the cell [76]. Acidification quickly inhibits cell growth and metabolism [77]. Therefore, the media must be buffered around the optimal pH for the microorganism to be investigated. For example in industrial yeast fermentations, it is relevant to maintain pH around 5.5. We compared the influence of 50 mM buffering salts on growth of TMB3001 in YP and in DM media (Figure 5). The suitability of citrate, citrate/phosphate, phosphate and phthalate to buffer the growth medium at pH 5.5 were compared.

YP had an inherent buffering capacity, while pH in non-buffered DM decreased to 2.5 when maximum OD_620 _was reached. The presence of citrate and citrate/phosphate severely inhibited growth in YP, whereas the inhibition was somewhat less severe in DM. With three carboxyl groups, citrate is a chelating compound and complexes with trace elements in YP. In DM, where the concentration of trace elements has been enhanced (Table 1), the inhibition of citrate was less severe. Phthalate showed the best buffering capacity, however, the price of this buffering compound may limit its use in large amounts. With phosphate buffer, pH of DM medium dropped to around 3 in the late stationary phase, but no growth inhibition was observed. Thus depending on the scale of strain screening either phthalate or phosphate buffer should be used for yeast development work.

### Auxotrophic markers: pros and cons

The construction of recombinant strains requires selectable marker genes for efficient detection and selection of transformed cells. For *S. cereviciae*, mutant and deletion strains having one or several auxotrophic requirements are the most commonly used tools in the development of recombinant strains [78]. The use of auxotrophic mutants relies on the assumption that complementing auxotrophy by plasmid expression makes the strain equivalent to its prototrophic counterpart. However, this is not always the case, as was shown for strains carrying the *LEU2 *gene on a multicopy plasmid [34].

In addition to the auxotrophic markers used for plasmid retention, uncomplemented auxotrophic mutations often remain present in the transformed yeast strains, requiring the addition of the necessary amino or nucleic acids to the cultivation medium. The use of such auxotrophic strains has recently been critically reviewed [36]. Based on the complications involved in translating experimental data obtained with auxotrophic strains into quantitative physiological data, the author concluded that auxotrophic strains should be avoided unless auxotrophy itself was under investigation. A solution to this problem is genetic complementation of the remaining auxotrophic markers, which is quite simple (see e.g. [32,37]) and recovers the prototrophic genotype.

Uncomplemented auxotrophic mutations can also affect production levels of recombinant proteins [36]. This was recently confirmed when growth and extracellular protein production were compared for an auxotrophic and a prototrophic *S. cerevisiae *strain expressing the *Trichoderma reesei *β-1, 4-xylanase *XYN2 *gene [37]. Only excessive amino acid supplementation allowed the auxotrophic strain to produce the heterologous protein at levels comparable to the prototrophic strain. Other studies have confirmed that excessive auxotrophic markers in transformed *S. cerevisiae *strains often result in overconsumption of the required metabolite and decreased growth, protein production and genetic stability [9,34,45,79-84]. These studies clearly demonstrated that physiological data obtained with auxotrophic strains have to be evaluated with great caution and should not form the basis for future strain design strategies.

### Media requirements/supplements for heterologous protein production

Expression of proteins is an inherent strategy of metabolic engineering, whether it is performed for the production of the protein itself or for redirecting a metabolic pathway. It was early observed that high-level protein expression influenced cell physiology of both prokaryotic and eukaryotic microorganisms (lit. reviewed in [37,38,85]). The most prominent effect was reduced cell growth. The phenomenon was named "metabolic" [53,86] and later "protein" [40,87] burden since it was discriminated from the influence of the catalytic activity of the protein [87]. The magnitude and the cause of the protein burden were estimated by comparing recombinant strains of *S. cerevisiae *expressing *T. reesei *β-1, 4-xylanase encoded by the *XYN2 *gene [85]. Both the introduction of the glycolytic promoter without the structural gene, and the structural gene itself, exercised a metabolic burden on the host. The reduction of the maximum specific growth rates, the biomass yields and the specific glucose consumption rates were much larger than expected from the amount of heterologous protein produced [85]. When the cultivation medium was supplemented with a balanced mixture of preferred amino acids (Ala, Arg, Asn, Glu, Gln and Gly) or succinate, the detrimental metabolic effect could partially be relieved [38]. Amino acids enhanced cell growth and heterologous protein production, which supported the observation that recombinant yeast expressing heterologous proteins experience depletion of amino acids and biosynthetic precursors [88].

The latter observation was the basis for genome-wide transcription analysis of two isogenic strains of *S. cerevisiae *harboring either a multicopy plasmid with the *T. reesei XYN2 *gene under control of the *S. cerevisiae PGK1 *promoter [58] or the plasmid with neither the structural gene nor the promoter. Transcription data (available at [89,90]) are summarized in Table 3. Transcriptional profiles during the expression of the heterologous xylanase strongly resembled severe amino acid limitation resulting in up-regulation of amino acid transport and synthesis, complemented by the induction of the general stress response and respiration, and the repression of ribosomal and glycolytic gene expression. The transcriptional response to heterologous xylanase expression thus closely resembled the stringent stress response, apparently due to amino acid limitation [38,91,92]. Similar stringent stress response has been reported for strains of the bacterium *E. coli *overproducing heterologous proteins. The *E. coli *stringent stress response normally involves the repression of ribosome synthesis and the derepression of respiration, amino acid uptake and amino acid biosynthesis due to nutrient limitation [93]. The stringent stress response in *S. cerevisiae *has previously been associated with nitrogen limitation and a nutritional downshift [94-97]. The fact that amino acid supplementation of the cultivation medium also improved heterologous protein production under control of an oxygen regulated promoter in the yeast *Pichia stipitis *[39] seems to further support that the induction of a nitrogen starvation response due to heterologous protein expression is general.

The choice of nitrogen source in cultivation media for the production of heterologous proteins is crucial as has been amply illustrated by observations with various complex nitrogen sources for industrial protein production. Inconsistency in complex components such as yeast extract can limit the reproducibility of industrial fermentation performance, resulting in 2–3 fold differences in heterologous protein production levels [9]. For industrial production, the proteins of complex cheese whey can be hydrolysed by proteases to allow for utilization by micro-organisms [98], which has been shown to improve the heterologous protein production compared with mineral medium [99,100]. However, other reports have indicated slower growth and lower production of heterologous proteins in cheese whey compared to mineral medium containing lactose [101].

The presence of nitrogen components in cultivation media may also be important to protect heterologous proteins in the extracellular medium from proteolysis. Extracellular proteolysis of heterologous proteins is affected by nutritional conditions, and may increase due to glucose exhaustion or carbon starvation [45,102,103]. The addition of complex nitrogen sources, such as casamino acids, peptides, amino acids, skim milk or bovine serum albumin has been shown to decrease the degradation of the heterologous proteins by *S. cerevisiae *and *P. pastoris*, probably by providing large amounts of protein substrate or reducing the production of extracellular proteases [81,104-110]. Addition of the amino acids arginine and lysine to cultures of *S. cerevisiae *in defined medium has decreased proteolysis of extracellular recombinant proteins, most likely due to the inhibition of proteolytic enzymes specific for peptide bonds including basic amino acid [111,112]. Buffering the cultivation medium to a pH where protein degradation is minimized can also reduce the breakdown of heterologous proteins [104,107,113-116].

Particular carbon sources may also be required to support heterologous protein production during specific growth phases. The production of recombinant antigens during gluconeogenesis in *S. cerevisiae *required additional medium components such as lactate and trehalose to ensure sufficient availability of metabolic energy [9]. In addition, too high concentrations of salts may reduce heterologous protein production [117].

For industrial production of high-value heterologous proteins such as bio-pharmaceuticals, the higher costs associated with the use of a defined mineral medium may be justified on the basis of increased reproducibility, productivity, and requirements for regulatory approval [45]. For both defined and complex media, the negative effect of nutrient limitations can be minimized by optimizing the concentrations of the medium components. This should preferably be done by the response surface methodology (see e.g. [118,119]). Such an empirical procedure is required separately for each heterologous protein, for each of which the optimal medium compositions may differ substantially.

### Industrial media for bulk bio-commodity production

Media components have a very strong impact on economics of industrial fermentation processes and can account for up to 30% of the total production cost [120,121]. Large scale production of cheap commodities such as fuels, chemicals and materials requires very cheap raw material [14,15]. Such processes use by-products from the agricultural, forestry and chemical industry as carbon and nitrogen sources. Carbon sources include sugar beet and sugar cane molasses, residues from sugar production, spent sulphite liquor (SSL) from the paper pulping industry, and cheese whey from the dairy industry. Spent yeast biomass can be processed to obtain valuable medium supplements (see e.g. [120,121]) and may serve as replacement for the more expensive yeast extract. A frequently used nitrogen source in industrial fermentation processes is corn steep liquor formed during starch production from corn [14,15].

Economic constraints in large-scale industrial processes rigorously limit the utilization of additives and pretreatments prior to cultivation. For the production of cell mass and ethanol using molasses and SSL, salts of ammonia are often the only additive providing the desired pH, a nitrogen source and possibly phosphate. Production of cell mass and ethanol are usually performed under non-sterile conditions [73] at a low pH, which allows yeast to grow while the growth of contaminating microorganisms is inhibited. It is obvious that production strains working efficiently in such media are widely different from laboratory-strains initially used to develop novel metabolic traits. The environmental constraints of industrial fermentation media will be summarized under the following headings: (*i*) multiple sugar substrates to be converted into the final product; (*ii*) by-product formation; (*iii*) nutrient limitation; and (*iv*) inhibitors.

#### (i) Multiple substrates

In addition to easily metabolized sugars, industrial substrates may also contain a mixture of more unusual sugars. For example, beet and cane molasses contain galactose, raffinose, and melibiose; starch derived substrates contain maltose; and hemicellulose-derived substrates contain galactose, mannose, xylose and arabinose. For maximum process economy all sugars should be converted to the desired product. The simultaneous presence of multiple sugars in the industrial media may pose limitations such as incomplete substrate utilization and inhibition of sugar utilization pathways. Some sugars such as galactose [122] and mannose are metabolized by *S. cerevisiae*, whereas the utilization of other sugars such as raffinose [123-125], melibiose [126], xylose [127] and arabinose [30,32] requires that a new metabolic pathway is genetically introduced. In addition, "natural" sugar utilization by *S. cerevisiae *is governed by carbon catabolite repression [128] and pathway induction [129], such that glucose and mannose are utilized first and other sugars are consumed only when these carbon sources are depleted. To circumvent this phenomenon, recombinant yeasts engineered in key signaling elements of the carbon catabolite repression cascade have been developed [130,131], which resulted in enhanced total sugar consumption rate. However, the feasibility of such engineered strains in industrial environments remains to be demonstrated. Carbon catabolite repression can also be overcome by using fed-batch fermentation regimes [132], which are easily applicable in industrial processes.

#### (ii) By-products

Glycerol is formed in relatively small amounts during anaerobic ethanolic fermentation [133]. However, considering the scale of ethanol production this unwanted by-product represents product losses in the million € range. Glycerol production during ethanolic fermentation is a consequence of surplus NADH formation in biosynthetic reactions [134-136]. During anaerobic growth of *S. cerevisiae *in the absence of an active respiratory pathway, biosynthetic NADH can only be oxidized through the reduction of dihydroxyacetone-phosphate to glycerol 3-phosphate, which ultimately leads to glycerol secretion. It was experimentally demonstrated that glycerol secretion is directly linked to amino acid synthesis in *S. cerevisiae *[135], Glycerol production was reduced when ammonium in the cultivation medium was substituted with amino acids. However, amino acid supplementation of industrial substrates for large-scale ethanol production is presently not considered economically viable even with relatively cheap protein hydrolyzates such as yeast extract and peptone.

Reducing glycerol formation during ethanolic fermentation has also been approached with metabolic engineering strategies. Bacteria harbor transhydrogenase enzymes, which convert NADH into NADPH in response to cellular requirements. Attempts to express these enzymes in *S. cerevisiae *have met with limited success [137,138]. Instead, endogenous redox reactions of the ammonia and amino acid metabolism in *S. cerevisiae *have been engineered to create artificial transhydrogenase functions [139,140]. In anaerobic cultivation, ethanol formation increased at the expense of glycerol formation in the engineered strains [138]. The use of such engineered strains in industrial applications remains to be demonstrated.

Anaerobic fermentation of xylose results in xylitol formation as a consequence of the difference in co-factor preference in the xylose reductase and xylitol dehydrogenase reactions, respectively (reviewed in [127]). Xylose reductase can use both NADPH and NADH as cofactor, whereas xylitol dehydrogenase exclusively uses NAD+. Xylitol is secreted and lost for ethanol production as a consequence of intracellular NAD+ depletion. Several strain design strategies have been explored to increase ethanol formation during xylose fermentation including modulations of intracellular co-factor availability [141-143] and expression of a mutated xylose reductase with reduced affinity for NADPH [144]. None of the engineered strains have so far been reported to be exposed to an industrial substrate.

An unexpected, yet fully explainable observation is that industrial cultivation media sometimes decrease unwanted by-product formation. For natural xylose fermenting yeast it was recognized that the reduction of an external electron acceptor such as acetoin provided NAD+ for the xylitol dehydrogenase reaction, which prevented xylitol formation [145-147]. For recombinant *S. cerevisiae *the same phenomenon was quantified with metabolic flux analysis [148]. The fact that recombinant laboratory strains of *S. cerevisiae *produced more ethanol in a lignocellose hydrolysate was interpreted in terms of lignocellulose derived components acting as external electron acceptors ([149]; see further discussion below).

Ethanol is an unwanted by-product in baker's yeast production [150,151]. Baker's yeast is industrially produced using a fed-batch regime, where the carbon substrate is fed into the production vessel at a rate which prevents "overflow" metabolism at the level of pyruvate and thus limits ethanol formation [152]. Since *S. cerevisiae *is also used for large-scale heterologous protein production [153], the unwanted ethanol formation during cell mass production has been approached by genetic engineering. The affinity of a *S. cerevisiae *hexose transporter has been reduced by gene shuffling [154] as well as by chemostat selection [155]. For both yeast strains, reduced ethanol formation during batch growth at high glucose concentration could be demonstrated as a consequence of the reduced glucose uptake rate. Such engineered strains are advantageous in the field of heterologous protein production, but it is more doubtful whether such strains ever can replace the simple fed-batch fermentation regime in baker's yeast production. For baker's yeast the characteristics of the product inherently include efficient carbon dioxide formation under non-growing oxygen limited conditions and it has not yet been demonstrated that this feature remains un-impaired in strains with reduced glycolytic rate.

#### (iii) Nutrient limitation

Nutrient limitation and starvation with respect to industrial yeast fermentation has mainly been discussed in relation to the classical processes of beer, wine and baker's yeast production. It may lead to "stuck" fermentation, which translates into large economic losses to the industry. New insight into the molecular mechanisms of nutrient limitation and starvation [156] makes this field of research develop rapidly [156-158]. Whereas media and strain modification in the production of beer, wine and baker's yeast may be limited by legislation and the final organoleptic quality of the product, the large scale fuel, materials and chemical industry is limited by economic constraints. Therefore, it remains to be demonstrated that recent research on nutrient starvation in yeast can be translated into novel fermentation strategies and novel industrial fermentation substrates. In ethanol production for the fuel and chemical markets, one rather relies on natural strain isolates, which have fully adapted to nutritional variation.

#### (iv) Inhibitors

Fermentation substrates for the production of fuels, materials and chemicals will be produced from lignocellulosic raw materials rather than from starch and sugar. The liquefaction of lignocellulose inherently leads to the formation of weak acids, furan derivatives and phenolic derivatives [74,159]. It is well known that weak acids can act as uncouplers and stimulate ethanol production [68,160]. Similarly, furan and phenolic compounds often appear carbonylated and as such function as external electron acceptors, which in the case of xylose fermentation is beneficial for ethanol formation (see (ii) By-products; [148,149]). However, the beneficial effect of these compounds is strongly concentration dependent and they more often act synergistically to inhibit yeast fermentation [159]. Therefore, the majority of reports on the fermentation of hydrolysates derived from lignocellulosics deal with the inhibitory characteristics of such fermentation substrates.

Lignocellulose hydrolysates have to be detoxified prior to fermentation [161,162]. However, the detoxification adds cost to the process [163] and should therefore be avoided. An elegant solution was demonstrated by applying a fed-batch regime to the fermentation of lignocellulosic hydrolysate [164]. Numerous yeast strains have been evaluated for their ability to ferment non-detoxified lignocellulose hydrolysate [165-169] The results of these studies are not always coherent, which reflects the profound influence of fermentation conditions such as media composition, oxygenation and fermentor set-up. However, it emerges that most laboratory strains used in the early stages of strain development cannot be used for an industrial raw material such as hydrolyzed lignocellulosics, whereas strains isolated from industrial environments generally perform much better. This was illustrated by comparing three different recombinant *Saccharomyces *strains expressing the *XYL1*, *XYL2 *and *XKS1 *genes for their ability to grow and ferment sugars in non-detoxified northern spruce hydrolysates (NSH). The laboratory strain TMB3001 [62] could not even tolerate 20% NSH, whereas the industrial strain TMB3400 [29] could grow in 33% NSH after supplementation with yeast extract. TMB3006 [170] derived from an acetic acid-tolerant *Saccharomyces *industrial strain isolated from a continuous spent sulfite liquor fermentation plant [171], could sustain growth in 40% NSH.

Strains tolerant to industrial media can be further improved by evolutionary engineering [18-20]. After exposing TMB3006 to continual selection to a NSH gradient of 40 – 70%, a NSH-adapted strain was obtained that could sustain growth in 70% NSH. This strain could be maintained in steady state at a dilution rate of D = 0.1 h^-1 ^with an ethanol yield of 0.41 g/g on consumed glucose, which illustrated the importance of strain background to achieve the necessary robustness to ferment harsh sugar syrups, such as NSH.

### Concluding remarks

The current literature on media composition in different stages of strain development for large scale industrial yeast fermentation has been summarized with a view that media composition is an integral part of strain development. In particular the final industrial environment must be carefully considered throughout the strain development process in order to assure the successful introduction of novel engineered strains into large-scale industrial processes.

## Figures and Tables

**Table 1:** Media composition

**Components (g l^-1^)**	**YP**	**DM**	**YNB**	**SC (YNB+Suppl)**
Yeast extract	10	-	-	-
Peptone	20	-	-	-
(NH_4_)_2_SO_4_	-	5	5	5
KH_2_PO_4_	-	3	1	1
MgSO_4_·7H_2_O	-	0.5	0.5	0.5
NaCl	-	-	0.1	0.1
				
**Vitamins (mg l^-1^)**				

Biotin	-	0.05	0.002	0.002
D-Pantothenic Acid	-	1	0.4	0.4
Nicotinic Acid	-	1	0.4	0.4
myo-Inositol	-	25	2	2
Thiamine	-	1	0.4	0.4
Pyridoxine	-	1	0.4	0.4
p-Aminobenzoic Acid	-	0.2	0.2	0.2
Riboflavin	-	-	0.2	0.2
Folic Acid	-	-	0.002	0.002
				
**Trace Elements (mg l^-1^)**				

H_3_BO_3_	-	1	0.5	0.5
CuSO_4_·5H_2_O	-	0.3	0.04	0.04
KI	-	0.1	0.1	0.1
Na_2_MoO_4_·2H_2_O	-	0.4	0.2	0.2
ZnSO_4_·7H_2_O	-	4.5	0.4	0.4
FeSO_4_·7H_2_O	-	3	-	-
FeCl_3_·6H_2_O	-	-	0.2	0.2
MnCl_2_·2H_2_O	-	1	-	-
MnSO_4_·4H_2_O	-	-	0.4	0.4
EDTA	-	15	-	-
CoCl_2_·6H_2_O	-	0.3	-	-
CaCl_2_·2H_2_O	-	4.5	100	100
				
**Supplements (mg l^-1^)**				

Adenine (hemisulfate salt)	-	-	-	40
L-arginine (HCl)	-	-	-	20
L-aspartic acid	-	-	-	100
L- glutamic acid (hemisulfate salt)	-	-	-	100
L-histidine	-	-	-	20
L-leucine	-	-	-	60
L-lysine (mono-HCl)	-	-	-	30
L-methionine	-	-	-	20
L-phenylalanine	-	-	-	50
L-serine	-	-	-	375
L-threonine	-	-	-	200
L-tryptophan	-	-	-	40
L-tyrosine	-	-	-	30
L-valine	-	-	-	150
Uracil	-	-	-	20

**Table 2:** Growth on glucose and xylose, respectively, and xylose uptake rate for recombinant xylose-utilizing *S. cerevisiae *strains, TMB 3001 [62] and TMB 3400 [29]. Cells were pre-grown (16 h) in DM medium with 20 g l^-1 ^glucose, harvested by centrifugation and washed three times before inoculation at 0.01 g l^-1^. Aerobic growth: 100 ml medium in 1000 ml baffled E-flask at 180 rpm. Oxygen-limited growth: 78 ml medium including 1.25 ml l^-1 ^Ergosterol/Tween^® ^80 in 80 ml flask with rubber septum and cotton-filled needle for CO_2 _outlet, stirring speed 150 rpm by a 2 cm magnetic stirrer bar. Media containing 20 g/l glucose and 20 g/l xylose, respectively, were buffered to pH 5.5 with 50 mM phthalate and the temperature was set at 30°C.

		**Aerobic**				**Ox-lim**			
		**YP**	**DM**	**YNB**	**SC**	**YP**	**DM**	**YNB**	**SCP**
**Glucose μmax (h^-1^)**	**TMB 3001**	0.56	0.42	0.42	0.45	0.40	0.32	0.32	0.32
	**TMB 3400**	0.59	0.43	0.45	0.49	0.47	0.36	0.35	0.38

**Xylose μmax (h^-1^)**	**TMB 3001**	0.32	0.14	0.14	0.14	0.009	0.006	0.006	0.006
	**TMB 3400**	0.40	0.26	0.27	0.30	0.021	0.012	0.013	0.015

**Vol. Xylose rate (g l^-1 ^h^-1^)**	**TMB 3001**	0.10	0.013	0.016	0.018	0.025	0.013	0.013	0.013
	**TMB 3400**	1.13	0.56	0.31	0.39	0.12	0.09	0.09	0.09

**Table 3:** Summary of overall transcriptional changes in cellular processes of xylanase-producing *S. cerevisiae *compared to reference strain

**Cellular Process**	**Order of magnitude change**
μ_max _(measured rate)	**↓↓ **[85]
Glucose uptake rate (measured rate)	**↓↓ **[85]
Glycolysis	**↓**
Amino acid requirement	**↑↑**
NH_4_^+ ^utilisation	**↓**
Respiration and TCA	**↑**
Iron uptake	**↑**
Transcription machinery	**↑**
Protein synthesis	**↓**
General stress response	**↑↑**
